# Quality of hospital care for sick newborns and severely malnourished children in Kenya: A two-year descriptive study in 8 hospitals

**DOI:** 10.1186/1472-6963-11-307

**Published:** 2011-11-11

**Authors:** David Gathara, Newton Opiyo, John Wagai, Stephen Ntoburi, Philip Ayieko, Charles Opondo, Annah Wamae, Santau Migiro, Wycliffe Mogoa, Aggrey Wasunna, Fred Were, Grace Irimu, Mike English

**Affiliations:** 1 KEMRI/Wellcome Trust Research Programme, Nairobi, Kenya; 2 Ministry of Public Health and Sanitation, Nairobi, Kenya; 3 Ministry of Medical Services, Nairobi, Kenya; 4 Department of Paediatrics, University of Nairobi, Nairobi, Kenya; 5 Department of Paediatrics, University of Oxford, Oxford, UK

**Keywords:** newborns, child malnutrition, quality of health care

## Abstract

**Background:**

Given the high mortality associated with neonatal illnesses and severe malnutrition and the development of packages of interventions that provide similar challenges for service delivery mechanisms we set out to explore how well such services are provided in Kenya.

**Methods:**

As a sub-component of a larger study we evaluated care during surveys conducted in 8 rural district hospitals using convenience samples of case records. After baseline hospitals received either a full multifaceted intervention (intervention hospitals) or a partial intervention (control hospitals) aimed largely at improving inpatient paediatric care for malaria, pneumonia and diarrhea/dehydration. Additional data were collected to: i) examine the availability of routine information at baseline and their value for morbidity, mortality and quality of care reporting, and ii) compare the care received against national guidelines disseminated to all hospitals.

**Results:**

Clinical documentation for neonatal and malnutrition admissions was often very poor at baseline with case records often entirely missing. Introducing a standard newborn admission record (NAR) form was associated with an increase in median assessment (IQR) score to 25/28 (22-27) from 2/28 (1-4) at baseline. Inadequate and incorrect prescribing of penicillin and gentamicin were common at baseline. For newborns considerable improvements in prescribing in the post baseline period were seen for penicillin but potentially serious errors persisted when prescribing gentamicin, particularly to low-birth weight newborns in the first week of life. Prescribing essential feeds appeared almost universally inadequate at baseline and showed limited improvement after guideline dissemination.

**Conclusion:**

Routine records are inadequate to assess newborn care and thus for monitoring newborn survival interventions. Quality of documented inpatient care for neonates and severely malnourished children is poor with limited improvement after the dissemination of clinical practice guidelines. Further research evaluating approaches to improving care for these vulnerable groups is urgently needed. We also suggest pre-service training curricula should be better aligned to help improve newborn survival particularly.

## Background

More than 7 million children die each year worldwide[1], most from preventable causes and almost all are in poor countries[1,2]. Of these, an estimated 3 million babies die in the first 4 weeks of life, the majority in the first week, accounting for 41% of mortality under 5 years [1,2]. The Millennium Development Goal 4 for child survival (MDG-4) is therefore unlikely to be met in many countries without a substantial reduction in neonatal mortality [1,2]. Several interventions to help reduce neonatal mortality should be delivered or supported by rural hospitals. These include keeping babies warm, provision of oxygen, assisted feeding and administration of antibiotics [3-5]. From a system perspective, these elements of care have much in common with those required for another highly vulnerable patient population, those with severe acute malnutrition, whose care is organized by the World Health Organization (WHO) into 10 steps [6,7]. However, in reports from African hospital settings mortality in both these vulnerable groups is often at least 20% [8-10]. At least for malnutrition much of this high inpatient mortality has been attributed to provision of inappropriate and poor quality clinical care [8,11].

Given the high mortality of these conditions and the development of packages of interventions that provide similar challenges for service delivery mechanisms we set out to explore how well such services are provided in Kenya, conducting a descriptive study while undertaking a parent multi-centre intervention study [12-14]. Our aim, using a simple set of indicators, was to provide basic data on quality of care helping to define the level of need for improvement and to inform design of future intervention studies.

## Methods

### Study design and participants

The study was conducted in eight district hospitals, initially identified on the basis of five district-specific criteria (additional file 1) and hospital workload statistics requiring a minimum of 1000 paediatric admissions and 1200 deliveries per year [12,13]. The location of the hospitals and basic facility characteristics are described in detail elsewhere[13]. In brief the parent study was a parallel group, controlled intervention study with hospitals randomized into full (n = 4) or partial (referred to as control, n = 4) intervention groups [13]. The parent study design, the process of developing the intervention and the contexts within which the study intervention was delivered are described in detail elsewhere[13].

Data reported here were collected on admission episodes for sick newborns aged 0-7 days and malnourished children aged 6-59 months by retrospectively examining records during hospital surveys conducted at baseline and subsequently over a period of 1.5 years. These data, collected as part of an exploratory sub-study, were aimed at: i) Examining the availability of routine data at baseline and their value for routine morbidity, mortality and quality of care reporting, ii) gaining preliminary data on quality of care and initial insights into the degree to which provision of new job aides and guidelines might influence the quality of care.

### Study intervention

The intervention was designed by the research team in collaboration with the Ministry of Health. The research team visited all sites before randomization and conducted a comprehensive baseline survey (survey 1). We employed a multi-faceted intervention aiming to improve paediatric care in Kenyan district hospitals through implementing best-practice guidelines for common severe childhood illnesses. The intervention's aspects relevant to the sub-component of the study examining care of newborns and cases of severe malnutrition included: a) provision to all hospitals of evidence based clinical practice guidelines booklets and wall-charts, these included advice on treatment for sick newborns and severely malnourished children including drug dosage tables and feed volume charts; b) provision to all hospitals of standard neonatal and pediatric admission record forms; and c) a 5.5 day training, spanning care of the sick newborn and child in intervention hospitals [12], or a 1.5 day guideline seminar on the same topics but using lectures only provided to control hospitals. Supervision, feedback and facilitation, provided to intervention hospitals only, [12,14] largely focused on availability of hospital resources for newborn and paediatric inpatient care [15] and the quality of care provided to infants and children on paediatric wards with pneumonia, diarrhea, and malaria. Therefore, although the term control hospital is used in this report both groups received interventions after baseline that varied in intensity. The primary study endpoints were largely based on the quality of care provided to children with pneumonia, diarrhea, and malaria and are reported elsewhere [16].

### Sample size and data collection

We were aware that considerable problems have existed in identifying neonatal and malnutrition case records in Kenyan hospitals [17]. Our aim was therefore to identify 30 records for each from every hospital during surveys at baseline (survey 1) and at approximately: 5-6 months post-intervention (survey 2), 11-12 months post-intervention (survey 3) and 18 months post-intervention (survey 4). Data thus represent a convenience sample, with retrieval of records beginning with those nearest in time to the survey and extending back in time until 30 records had been identified or until a maximum period of 6 months prior to the survey had been exhausted. Data collection was performed by a trained survey team abstracting information from case records into structured questionnaires and used strict, standard operating procedures. A 10% random sample of records was independently abstracted by a team supervisor and results of the two procedures compared. Agreement rates were consistently greater than 95%. All data were subsequently double entered and verified using Microsoft Access^®^.

### Indicators

Based on the key messages in the training and guidelines provided after baseline surveys and using these as reference standards we developed quality of care indicators before the start of the study for both patient groups. These are shown in additional file 2[7,18].

### Analysis

We present results at baseline for our set of key indicators for the 8 hospitals individually and as pooled, weighted group summaries. Because there are a relatively limited number of observations from each survey we pooled post-baseline data across surveys 2 to 4 within hospitals. We then calculated weighted group summaries (intervention, control and all hospitals) to ensure each site contributed equally to any overall group measure. We present data on intervention and control hospital groups separately post-baseline as they were treated differently during the intervention period. However, we do not report 95% confidence intervals around summary estimates since these are not based on a random sample of all records and would suggest spurious precision. Thus, we suggest findings of this study are interpreted as illustrative only with post-randomization data in the intervention and control groups primarily providing an initial indication of care under better than typical conditions over a 1.5 years period. All analyses were undertaken using Stata v11 (StataCorp, Texas, USA). Scientific and ethical approval for the study was obtained from the Kenya Medical Research Institute National Scientific and Ethical Review Boards and study hospital management teams provided assent for inclusion before baseline surveys.

## Results

### Newborns

We anticipated evaluating 960 inpatient neonatal admission episodes but were able to retrieve a total of 798 (83%) records for these analyses with some imbalance between sites. Of these 52% (418/798) were from hospitals in the intervention arm. Based on hospital clinicians' diagnoses, and assuming the first recorded was the primary diagnosis, available data suggest that prematurity/very low birth weight (313 (39%)), birth asphyxia (212 (27%)), neonatal sepsis (131 (16%)) and neonatal respiratory distress syndrome (89 (11%)) were the commonest major causes of admission. The overall inpatient neonatal mortality, where data on outcome were available, was 38% (241/639, additional file 3). The mortality rates for low-birth weight infants (<2000 gm) and infants with estimated gestation ≤35 weeks were 51% (111/219) and 54% (76/140) respectively. Where data are available they indicate that 28% (196/698) of neonates were born outside the hospital, with two sites contributing more than 50% of these out-born infants.

#### Baseline findings

At baseline 189 records were available and examined across the 8 hospitals. The median assessment score we used was an index based on documentation of core clinical signs and symptoms, it had a range from 0 (documentation completely absent) to 28 (perfect documentation). A median score of 2 (inter-quartile range, IQR, 1-4) was observed at baseline, indicating extremely poor routine clinical documentation of admission episodes. The median (IQR) crystalline penicillin dose for infants <7 days old (n = 30) was almost twice that recommended with 34% (15/44) of penicillin prescriptions being for an inappropriate four times daily regimen. Of gentamicin prescriptions with dose, weight and frequency documented, once daily dosing was rarely used (3/28, 11%). The total number of high doses for gentamicin was 25% (7/28). Vitamin K was prescribed in only 10% (19/189) newborns, all from only 3 sites. As documentation of clinical status was very poor it was not possible to establish which newborns met criteria indicating a probable need for oxygen and/or assisted feeding. Thus results for these indicators at baseline cannot be provided although 46% (87/189) had some form of feeding prescribed.

#### Post-intervention findings

Post-baseline 609 records were examined, hospitals contributed between 43 and 100 records from the 3 surveys. The NAR was used in 84% (278/332) records in intervention hospitals and 56% (156/277) records in control hospitals. The median (IQR) documentation score for the 28 core clinical signs was 26 (24 - 27) and 22 (17 - 26) in intervention and control groups respectively if the NAR was used, while the median (IQR) documentation score where the NAR was not used was 24 (12 - 26) and 2 (2 - 5) in intervention and control sites respectively.

Post-intervention the median (IQR) crystalline penicillin dose fell for newborns aged less than 7 days to about the recommended dose 113000 (100000 - 200000) iu/kg/day in intervention and control hospitals. Use of once daily gentamicin increased substantially to over 85% overall. The median dosage in mg/kg/day for children weighing 2 kg or more and <7 days old was close to that recommended in the guidelines (5 mg/kg/day), however, in the small number of newborns aged <7 days and weighing less than 2 kg where guidelines recommended 3 mg/kg/day the median dose of gentamicin was similar to that recommended for those weighing >2 kg (5 mg/kg/day). Overall proportions of neonates receiving a high dose of gentamicin were 46% (36/78) in the intervention arm and 29% (10/34) in the control arm. Vitamin K was prescribed in 51% (169/332) in the post-baseline period in the intervention arm and 37% (101/276) in the control arm.

Improved documentation post baseline, particularly in intervention sites, allowed examination of indicators of supportive care. Thus, 68% (227/332) and 29% (80/277) of neonates in intervention sites and control sites respectively had sufficient core clinical signs documented to estimate a sick newborn's likely need for oxygen. Of those likely to have required oxygen (additional file 2), the proportions of those prescribed it was half or less and prescriptions with flow-rate and route of oxygen administration clearly indicated were provided in less than 20%. Similarly the proportions of neonates estimated to require assisted feeding (additional file 2) and with no apparent contraindication were 69% (228/332) and 42% (115/277) in intervention and control sites respectively. Of these 16% (36/228) and 10% (12/115) had features to suggest a likely need for feeds in intervention and control sites respectively. The proportion actually prescribed feeds was similar in intervention and control hospitals at approximately 60% but fewer than 30% had a correct, age-specific, volume per kilogram body weight regimen prescribed.

### Malnutrition

In total 521 case records of children with a discharge or Health Information Systems (HIS) diagnosis of severe malnutrition were evaluated, with 79% (410/521) of these being in the post-intervention period. The median (IQR) age was 16 (11-24) months while the mean (SD) weight for age (WAZ) score was -2.4 (1.5) where weight was recorded (n = 394). The overall mortality was 25% (124/496) where outcome data were available. Co-existing co-morbidities included dehydration 29% (123/424), pneumonia 21% (91/424), diarrhea 7% (30/424), malaria 10% (43/424), anemia 6% (25/424), meningitis 2% (8/424) and others 7% (29/424). Additional file 4 shows the results from the severe malnutrition analysis.

#### Baseline findings

At baseline 111 case records were available for evaluation across the 8 hospitals. Assessment for clinical features of severe malnutrition was only documented in 5% (5/111), 41% (46/111) had a syndromic classification (marasmus, kwashiorkor or marasmic-kwashiorkor) while the remainder had only a general label of 'severe malnutrition'. The proportion of children with a documented random blood sugar (RBS) or who had dextrose given presumptively at admission, Step 1, was 4% (4/111) while temperature was documented for 25% (38/111) of the children. Of the 59% (66/111) children diagnosed as dehydrated none were correctly prescribed ReSoMal; 33% (22/66) were prescribed Oral Rehydration Solution (ORS) while 32% (21/66) were probably incorrectly prescribed intravenous fluids although shock was not documented. Crystalline penicillin in combination with gentamicin and metronidazole are recommended by Kenyan guidelines as empiric therapy of sick children with severe malnutrition admitted to hospital. At baseline penicillin and gentamicin were prescribed routinely to 66% (37/54) and 48% (30/57) in intervention and control hospitals respectively but very few children were prescribed metronidazole 4% (4/111). Of the four recommended micronutrients (vitamin A, zinc, folic acid and multivitamins) at least one was prescribed for 32% (35/111) children although none were prescribed vitamin A. At least one inappropriate drug (either iron or a de-worming agent that are not recommended before 7 days of admission) was prescribed in 28% (31/111) of cases. The proportion of children with feeds prescribed was 84% (93/111) however none of these were of the correct feed type and volume.

#### Post-intervention findings

At follow-up 410 records were available, with marked heterogeneity between sites for case numbers; in total 67% (275/410) were from intervention hospitals. There was considerable change in the use of standard syndromic classifications as diagnoses with 90% (246/275) and 82% (106/135) cases using classifications recommended by guidelines in intervention and control hospitals respectively. Of these 50% (134/275) and 35% (46/135) had a syndromic classification compatible with the clinical data recorded in intervention and control sites respectively.

Step 1 and 2 management of these cases were documented in 16% (79/410) and 56% (244/410) cases. Step 3 concerns management of dehydration, of 62% (252/410) children diagnosed as dehydrated only 1% (3/252) were correctly managed with ReSoMal; 27% (68/252) were prescribed ORS while 26% (66/252) were prescribed intravenous fluids despite none being in shock. Shock was diagnosed in 21% (53/252) and of these 21% (11/53) were prescribed the correct intravenous fluid.

Step 5 spans treatment of infections, about half had penicillin and gentamicin prescribed in the post-intervention period while metronidazole was prescribed a little more often than at baseline at 16% (45/275) in intervention hospitals and 9% (12/135) in control hospitals. Of those prescribed penicillin and gentamicin 54% (143/231) had the correct dose per kilogram per day. Step 6 advises appropriate micronutrient supplementation. At least one of the recommended micronutrients was prescribed to 75% (203/275) and 59% (88/135) cases in intervention and control hospitals respectively. Vitamin A supplementation improved from 0% at baseline to 51% (134/275) in intervention and 39% (58/135) in control sites. Of these over 80% had a correct dose for age. However, inappropriate drug prescription of either iron or a de-wormer persisted at 23% (62/275) and 43% (58/135) in intervention and control hospitals respectively.

Step 7, feeds were initiated on admission in 64% (267/410) of the children and although none of the feeds were of the correct type or volume at baseline, 61% (162/275) and 35% (53/135) were of the correct type in intervention and control hospitals post-baseline respectively. However, only small improvements were achieved in the correctness of feed volume. Overall correct initial feed therapy (correct feed type and volume combined) was 12% (33/275) and 4% (2/135) in intervention and control sites respectively.

## Discussion

We and others have previously reported poor quality of care for children with common, severe illnesses in low-income country hospitals [17,19-21]. Others have reported high specific case fatality rates in African hospitals for sick newborns and children with severe malnutrition [8-10]. Our baseline data indicate, despite these reports and the production of international guidelines aimed at reducing mortality, that care for such vulnerable groups remains poor, at least in rural Kenyan hospitals. The inadequacies in care, particularly that for neonates, at levels of the health system expected to provide expertise, supervision and leadership in support of primary care are of major concern and threaten progress towards the 4^th ^Millennium Development Goal.

Inadequacies or errors in care spanned use of basic antibiotics including penicillin and gentamicin. These drugs have been recommended for neonatal sepsis and severe malnutrition for decades and are used by health workers in probably tens of thousands of doses annually for neonatal sepsis in Kenya alone. Gentamicin is also being considered internationally for use by community health workers [22-24]. In the post-baseline period, after provision of guidelines, penicillin dosing improved. However, errors remained common for gentamicin prescriptions. Often it seemed errors were because prescribers were unaware of, or failed to consider, the specific dosing requirements for newborns in the first week of life. Poor prescribing was not confined to neonatal care however. Vitamin A, that can prevent blindness in children with severe malnutrition, was not prescribed to children at baseline although there was improvement in the post-baseline period. The results we report are a major concern given the critical importance now given to providing essential packages of care [5,25-28].

A report from these study hospitals at baseline on their readiness to provide newborn care indicates that they were ill prepared [15] and this may explain why care for newborns and those with malnutrition seemed to improve relatively little. However, in a recently published paper by Ayieko and colleagues[16] evaluating the parent study in these hospitals it was demonstrated that face to face feedback of performance, supportive supervision and provision of a local facilitator but no additional material resources did result in improvements of paediatric care and the readiness for providing care over the same period. Although the data for newborn care and care of those with malnutrition is poorer quality, the findings presented and experience during the intervention, suggest that a lower emphasis placed on supervision and feedback in these clinical areas may have contributed to their generally poorer performance improvement. In addition our experience suggests that health workers, who often receive as little as 2 weeks training in neonatal care, had limited knowledge and skills on the use of essential drugs or appropriate supportive care including feeding in vulnerable, often preterm, babies and children with severe malnutrition. Such shortcomings emanate from limitations in basic training for many health workers.

The other striking finding of our study is that it would appear almost impossible to make any reasonable assessment of neonatal care based on routine hospital records. Although we targeted a total of 960 records (i.e. 30 case records per site per survey) for each patient group record retrieval was as low as 7 newborn records per site per 6 months inter-survey period, a rate clearly inconsistent with likely workloads. Unfortunately even if records are present clinical documentation is often so poor that it is impossible to assess the adequacy of practice with even mortality data often missing. Such failings indicate an inability to assess quality of care, adequacy of intervention coverage and outcomes for newborns, all of which are essential to understanding health system performance.

Consistent with findings from other studies [29-36] and in the context of an intervention programme, there was marked improvement in case-specific documentation if a structured form was used, however it is difficult to quantify the extent to which this represented actual improvement of care - and this problem is an inevitable consequence of assessing quality from routine records in any setting. However, we are confident that changes in drug prescribing would lead to changes in actual care delivered and the changes observed in this area are similar to those in other areas [16].

Although standardized admission record forms may have the drawback of taking up more clinician time while staff become familiar with them the direct benefits of comprehensive evaluation, that may prompt more complete problem identification and consequently better treatment, would appear to outweigh this problem, particularly as there may be indirect benefits of decreased cost to the health system due to better use of resources. More efficient methods of clinical data collection could result from introduction of electronic medical records systems which have the further advantage of employing inbuilt clinical decision support systems and periodic reports. However such interventions will be limited by the availability of funds, expertise and electricity in many resource limited settings.

The data we report have several limitations. These include: a relatively small number of hospitals studied; the potential for selection bias at the point of record retrieval; possible ascertainment bias at the point of data abstraction from written records; misclassification of cases when populating quality indicators; and, the implicit assumption that the written records reflect actual practice. Despite this we feel our data still provide useful insight into the process and quality of care provided to newborns and severely malnourished children in Kenyan rural hospitals. Data of this type are rarely reported and we suspect, but cannot confirm, that data from Kenya may be indicative of problems present much more widely in low-income African settings given reports of poor quality of care for children in the region [37].

## Conclusion

In conclusion, our preliminary data indicate that many health workers at hospital level are unable to provide appropriately planned care for sick newborns or children with severe malnutrition. Inadequacies are seen in key tasks such as prescription of antibiotics and feeds even when resources are available and after provision of guidelines. Further, without improvements in hospital based information systems it will be impossible to know whether the frequent calls for deployment of and investment in interventions to improve care are being heeded. Problems exist despite a long history of development and formulation of international, and adapted national, guidelines. Future research should examine therefore how best to implement better care in rural hospitals, enabling them to serve their expected role in global efforts to reduce neonatal and child mortality in low-income settings such as Kenya.

## Competing interests

The authors declare that they have no competing interests.

## Authors' contributions

The idea for the study and its design were conceived by ME, with advice from AW, FW and AW. ME obtained the funding for this project. SN, JW, GI and ME provided and coordinated training and guideline dissemination; PA, NO, CO, SN, JW, GI, DG and ME were responsible for surveys, and data entry. DG, NO, and ME were responsible for data analyses and DG and ME prepared the initial draft manuscript. All authors reviewed the draft manuscript and provided input to and approval for the final version of the report.

## Pre-publication history

The pre-publication history for this paper can be accessed here:

http://www.biomedcentral.com/1472-6963/11/307/prepub

## Supplementary Material

Additional file 1 **Characteristics of study sites**. This file contains a table with the various characteristics of study hospitals considered before inclusion into the study. Click here for file

Additional file 2 **Performance indicators for newborns and severely malnourished children**. This file contains a table with the definitions for the various neonatal and severe malnutrition performance indicators that guided the analysis. Click here for file

Additional file 3 **Newborn quality of documentation and care by hospital and group**. This file contains a table with the results for the various indicators for neonates presented by hospital and group (intervention/control). Click here for file

Additional file 4 **Severe malnutrition quality of documentation and care by hospital and group**. This file contains a table with the results for the various indicators for severe malnutrition presented by hospital and group (intervention/control). Click here for file
