# Supplementary material for: Quality of hospital care for sick newborns and severely malnourished children in Kenya: A two-year descriptive study in 8 hospitals
Source: BMC Health Serv Res. 2011 Nov 11;11:307. doi: 10.1186/1472-6963-11-307 (PMC3236590; doi:10.1186/1472-6963-11-307)
Supplement: Additional file 1 — Characteristics of study sites. This file contains a table with the various characteristics of study hospitals considered before inclusion into the study. [file 1472-6963-11-307-S1.DOC]

**Additional file 1**: Characteristics of study sites

| **Hospital** | **Malaria transmission setting** | **Antenatal HIV prevalence High ≥10% Mod = 5–10%** | **No. of deliveries per year** | **No. of cots for neonatal admission[†](http://hinari-gw.who.int/whalecomwww.ncbi.nlm.nih.gov/whalecom0/pmc/articles/PMC2751740/table/tbl1/#tf1-1)** | **Infant mortality rate, per 1000** | **Catchment population with income below $2/day (%)** | **Paediatrician and Medical Officer Interns[‡](http://hinari-gw.who.int/whalecomwww.ncbi.nlm.nih.gov/whalecom0/pmc/articles/PMC2751740/table/tbl1/#tf1-2)** |
| --- | --- | --- | --- | --- | --- | --- | --- |
| H1 | Intense | High | 1750 | 3 | >100 | 50–70 | − |
| H2 | Highland | High | 4951 | 13 | ∼70 | 50–70 | + |
| H3 | Low | Moderate | 7500 | 9 | ∼40 | ∼35 | − |
| H4 | Arid | Moderate | 2080 | 4 | ∼70 | 50–70 | − |
| H5 | Intense | High | 1697 | 6 | >100 | 50–70 | − |
| H6 | Arid | Moderate | 1799 | 6 | ∼70 | 50–70 | − |
| H7 | Highland | High | 4235 | 14 | >100 | 50–70 | + |
| H8 | Low | Moderate | 3595 | 11 | ∼40 | ∼35 | − |

^†^This does not include capacity at other areas such as the paediatric wards where newborns are sometimes admitted.

^‡^A cadre of newly generated medical doctors attached to hospitals for 1 year for supervised practical experience.
