# Supplementary material for: Quality of hospital care for sick newborns and severely malnourished children in Kenya: A two-year descriptive study in 8 hospitals
Source: BMC Health Serv Res. 2011 Nov 11;11:307. doi: 10.1186/1472-6963-11-307 (PMC3236590; doi:10.1186/1472-6963-11-307)
Supplement: Additional file 2 — Performance indicators for newborns and severely malnourished children. This file contains a table with the definitions for the various neonatal and severe malnutrition performance indicators that guided the analysis. [file 1472-6963-11-307-S2.DOC]

**Additional file 2:** **Performance indicators for newborns and severely malnourished children**

|  | **Newborn indicators** | **Severe Malnutrition indicators based on WHO 10 steps** |
| --- | --- | --- |
| **Documentation** | 1. Uptake of the NAR as measured by the proportion of newborns clerked on a NAR in the post-baseline period 2. Completeness of case-documentation based on 28 core clinical signs and symptoms drawn from WHO and national recommendations and included on the NAR. | 1. Documentation of the recommended essential clinical signs required to make a syndromic diagnosis of severe malnutrition i.e. visible severe wasting and pitting edema allowing recognition of kwashiorkor, marasmic-kwashiokor and marasmus 2. Step 1, documentation of a random blood sugar test (RBS) done or intravenous dextrose given (presumptive management) where RBS test not available. 3. Step 2, documentation of temperature 4. Step 3, correctness of rehydration therapy; correct choice of intravenous fluid if in shock and correct choice of oral rehydration fluid (ReSoMal) if not in shock 5. Step 5, correctness of treatment practices evaluated by examining prescriptions for dose and frequency of gentamicin, penicillin and metronidazole and comparing these with weight and appropriate national guidelines (2005)[18]. 6. Step 6, documentation of prescription of at least one of the appropriate micronutrient (vitamin A, zinc, folic or multivitamin)    - Proportion with a Vitamin A prescription and correct dose as per age    - Proportion inappropriately given iron or a de-wormer at admission. 7. Step 7, correctness of feed therapy; correct feed type, volume and frequency appropriate for weight as per the recommended national guidelines (2005) |
| **Treatment**^†^ | 1. Correctness of treatment practices evaluated by examining prescriptions for dose and frequency of gentamicin and penicillin and comparing these with weight and post-natal age appropriate national guidelines.  - Over-dose was defined as 25% more than the recommended dose per kilogram body weight per day as per guidelines. |
| **Supportive care** | 1. **Oxygen**, correctness of prescription, defined as the proportion of neonates with any of cyanosis, grunting, severe chest-wall in-drawing or a respiratory rate >60 breaths per minute with oxygen prescribed with the correct route and rate as per guidelines. 2. **Feeds**, correctness of prescription (volume and frequency appropriate for post-natal age) as per guidelines in those unable to feed and weighing >1750 grams or weighing <1750 grams and unable to feed but without respiratory distress, and, finally; 3. Proportion of sick newborns aged <14 days given prophylactic Vitamin K at admission (Kenyan policy is for all newborns born in hospital or those admitted aged <14 days to receive such prophylaxis). |
