# Supplementary material for: Quality of hospital care for sick newborns and severely malnourished children in Kenya: A two-year descriptive study in 8 hospitals
Source: BMC Health Serv Res. 2011 Nov 11;11:307. doi: 10.1186/1472-6963-11-307 (PMC3236590; doi:10.1186/1472-6963-11-307)
Supplement: Additional file 3 — Newborn quality of documentation and care by hospital and group. This file contains a table with the results for the various indicators for neonates presented by hospital and group (intervention/control). [file 1472-6963-11-307-S3.DOC]

**Additional file 3: Newborn quality of documentation and care by hospital and group**

| **Hospitals** |  | **H1** | **H2** | **H3** | **H4** | **H5** | **H6** | **H7** | **H8** | **Intervention**^†^ | **Control**^†^ | **All pooled**^†^ |
| --- | --- | --- | --- | --- | --- | --- | --- | --- | --- | --- | --- | --- |
|  | | |  |  |  |  |  |  |  |  |  |  |
|  | Baseline, n | 17 | 14 | 31 | 24 | 10 | 14 | 40 | 40 | **86** | **103** | **189** |
|  | follow-up, n | 53 | 90 | 107 | 83 | 43 | 50 | 84 | 100 | **332** | **277** | **609** |
|  | NAR used, n | 45 | 56 | 102 | 75 | 28 | 27 | 5 | 96 | **278** | **156** | **434** |
| **Median documentation score (IQR) *range(0-28)*** | | | | |  |  |  |  |  |  |  |  |
|  | NAR not used | 13 (4 -22) | 26 (24 -27) | 25 (14 -27) | 4 (2-9) | 3 (2 - 15) | 6 (3 - 9) | 2 (1 -2) | 20 (17 - 22) | **24 (12 -24)** | **2 (2 - 5)** | **4 (2 - 18)** |
|  | NAR used | 26 (23 - 27) | 26 (24 -27) | 26 (23 -27) | 26 (24 -27) | 5.5 (2-16.5) | 22 (11 - 25) | 25 (24-25) | 24 (20-26 | **26 (24-27)** | **22 (17-26)** | **25 (22 - 27)** |
| **Documentation of gestation in weeks*** | | |  |  |  |  |  |  |  |  |  |  |
| follow-up | n (%) | 33 (62) | 44 (49) | 60 (57) | 59 (71) | 17 (40) | 20 (40) | 51 (61) | 40 (40) | **(60)** | **(45)** | **(53)** |
|  | median(IQR) | 34 (28 - 40) | 36 (30 - 39) | 36 (29 - 39) | 36 (30 - 40) | 30 (24 - 34) | 37 (32 - 40) | 36 (30 -38) | 39 (37 - 40) | **36 (30 - 40)** | **36 (31 - 40)** | **36 (30 -40)** |
| **Documentation of birth weight in grams** | | | |  |  |  |  |  |  |  |  |  |
| baseline | n (%) | 14 (82) | 10 (71) | 26 (84) | 11 (46) | 7 (70) | 12 (86) | 31 (80) | 40 (100) | **(71)** | **(84)** | **(77)** |
|  | median(IQR) | 1630 | 1830 | 1850 | 2500 | 1600 | 2550 | 2200 | 2230 | **2200** | | **2000** |
|  |  | (1100-1900) | (1500 -3100) | (1300 -2900) | (2000-3100) | (1100 -1850) | (1430 -3100) | (1600-3400) | (1600- 3350) | **(1500 - 3000)** | | **(1500 -3000)** |
| follow-up | n (%) | 47 (89) | 59 (66) | 96 (91) | 60 (72) | 36 (84) | 46 (92) | 79 (94) | 92 (92) | **(80)** | **(91)** | **(85)** |
|  | median(IQR) | 1900 | 1900 | 2350 | 2000 | 1280 | 2100 | 2000 | 2800 | **2000** | **2200** | **2100** |
|  |  | (1300-3000) | (1500-3100) | (1600-2900) | (1480-1780) | (1150 - 1500) | (1450 -3000) | (1500 -3000) | (2000-3200) | **(1500 - 2900)** | **(1500 - 3000)** | **(1500 -3000)** |
| **Mortality (%)** | |  |  |  |  |  |  |  |  |  |  |  |
|  | baseline | 8/10 (80) | 1/9 (10)* | 13/23 (57) | 11/24 (46) | 2/4 (50)* | 4/7 (57)* | 6/24 (20) | 5/36 (14) | **(48)** | **(35)** | **(42)** |
|  | follow-up | 15/31 (48) | 35/81 (43) | 36/104 (34) | 42/78 (54) | 0/6 (0)* | 19/41 (46) | 28/66 (42) | 16/88 (18) | **(45)** | **(35)** | **(36)** |
| **Vitamin K*** | |  |  |  |  |  |  |  |  |  |  |  |
|  | follow-up **(%)** | 9(17) | 40(44) | 78(74) | 42 (52) | 7(17) | 14 (28) | 5 (6) | 75 (75) | **(47)** | **(30)** | **(39)** |
| **Over-doses of penicillin *** | | |  |  |  |  |  |  |  |  |  |  |
|  | follow-up **(%)** | 11/21 (52) | 2/5 (40) | 12/37 (32) | 8/18 (44) | 1/1 (100) | 7/21 (33) | 1/2 (50) | 7/9 (78) | **(42)** | **(65)** | **(54)** |
| **Over-doses of gentamicin *** | | |  |  |  |  |  |  |  |  |  |  |
|  | follow-up **(%)** | 15/21 (71) | 0 | 14/36 (39) | 7/16 (44) | 0 | 7/20 (35) | 2/84 (2) | 1/10 (10) | **(51)** | **(16)** | **(25)** |

*Inadequate data at baseline

^†^weighted pooled group summaries

NAR – Newborn admission Record
