# Supplementary material for: Quality of hospital care for sick newborns and severely malnourished children in Kenya: A two-year descriptive study in 8 hospitals
Source: BMC Health Serv Res. 2011 Nov 11;11:307. doi: 10.1186/1472-6963-11-307 (PMC3236590; doi:10.1186/1472-6963-11-307)
Supplement: Additional file 4 — Severe malnutrition quality of documentation and care by hospital and group. This file contains a table with the results for the various indicators for severe malnutrition presented by hospital and group (intervention/control). [file 1472-6963-11-307-S4.DOC]

**Additional file 4:** **Severe malnutrition quality of documentation and care by hospital and group**

|  |  | **HM** | **GY** | **SI** | **TU** | **BA** | **LO** | **KK** | **NR** | **% intervention**^†^ | **% control**^†^ | **% all pooled**^†^ |
| --- | --- | --- | --- | --- | --- | --- | --- | --- | --- | --- | --- | --- |
| available cases (age 6-59 months; surveys 1-6) | | | |  |  |  |  |  |  |  |  |  |
|  | baseline | 16 | 2 | 13 | 23 | 3 | 25 | 18 | 11 | 54 | 57 | 111 |
|  | follow-up | 76 | 54 | 65 | 80 | 49 | 35 | 35 | 16 | 275 | 135 | 410 |
| assessment | **proportion assessed for severe wasting and edema** | | | | |  |  |  |  |  |  |  |
|  | baseline | 0(0) | 0(0) | 2(15) | 0(0) | 0(0) | 0(0) | 2(11) | 1(9) | **4** | **5** | **4** |
|  | follow-up | 67(88) | 54(100) | 65(100) | 74(93) | 22(45) | 24(69) | 20(57) | 13(81) | **95** | **59** | **79** |
|  |  |  |  |  |  |  |  |  |  |  |  |  |
| step 1 | **proportion with glucose measured/dextrose given** | | | | |  |  |  |  |  |  |  |
|  | baseline | 1(6) | 0(0) | 1(8) | 0(0) | 0(0) | 1(4) | 1(6) | 0(0) | **4** | **3** | **4** |
|  | follow-up | 9(12) | 18(33) | 32(49) | 10(13) | 6(12) | 2(6) | 2(6) | 0(0) | **27** | **6** | **16** |
|  |  |  |  |  |  |  |  |  |  |  |  |  |
| step 2 | **proportion with temperature documented** | | | |  |  |  |  |  |  |  |  |
|  | baseline | 1(6) | 0(0) | 1(8) | 7(30) | 1(33) | 24(96) | 3(17) | 1(9) | **11** | **39** | **25** |
|  | follow-up | 26(34) | 49(91) | 45(69) | 62(78) | 14(29) | 33(94) | 12(34) | 3(19) | **66** | **46** | **56** |
|  |  |  |  |  |  |  |  |  |  |  |  |  |
| step 5 | **proportion with crystalline penicillin and gentamicin prescribed** | | | | | | |  |  |  |  |  |
|  | baseline | 13(81) | 1(50) | 10(77) | 13(57) | 1(33) | 16(64) | 7(39) | 6(55) | **66** | **48** | **57** |
|  | follow-up | 42(55) | 33(61) | 52(80) | 32(40) | 33(67) | 14(40) | 15(43) | 10(63) | **58** | **53** | **56** |
|  | **proportion with correct dosage per kg/day for crystalline penicillin and gentamicin** | | | | | | |  |  |  |  |  |
| n=67 | baseline | 0(0) | 0(0) | 4(40) | 0(0) | 0(0) | 0(0) | 0(0) | 1(17) | **10** | **4** | **7** |
| n=231 | follow-up | 28(67) | 27(82) | 41(79) | 12(38) | 20(61) | 2(14) | 12(80) | 1(10) | **67** | **41** | **54** |
|  |  |  |  |  |  |  |  |  |  |  |  |  |
| step 6 | **appropriate micronutrient supplementation** | | | |  |  |  |  |  |  |  |  |
|  | baseline | 8(50) | 0(0) | 5(38) | 4(17) | 2(67) | 7(28) | 9(50) | 0(0) | **26** | **36** | **31** |
|  | follow-up | 50(66) | 44(81) | 60(92) | 49(61) | 40(82) | 11(31) | 32(91) | 5(31) | **75** | **59** | **67** |
|  | **inappropriate micronutrient supplementation** | | | |  |  |  |  |  |  |  |  |
|  | baseline | 5(31) | 1(50) | 3(23) | 6(26) | 0(0) | 8(32) | 5(28) | 3(27) | **33** | **22** | **24** |
|  | follow-up | 23(30) | 1(2) | 24(37) | 14(18) | 21(43) | 11(31) | 22(63) | 4(25) | **23** | **43** | **35** |
|  |  |  |  |  |  |  |  |  |  |  |  |  |
| step 7 | **feeds prescribed** | |  |  |  |  |  |  |  |  |  |  |
|  | baseline | 15(94) | 1(50) | 10(77) | 21(91) | 3(100) | 17(68) | 16(89) | 10(91) | **78** | **87** | **83** |
|  | follow-up | 54(72) | 39(72) | 51(78) | 40(50) | 37(76) | 16(46) | 23(66) | 8(50) | **68** | **60** | **64** |
|  | **correct feed type*** | |  |  |  |  |  |  |  |  |  |  |
|  | follow-up | 44(58) | 36(67) | 50(77) | 32(40) | 22(45) | 9(26) | 20(57) | 2(13) | **61** | **35** | **48** |
|  | **correct feed volume*** | |  |  |  |  |  |  |  |  |  |  |
|  | follow-up | 11(14) | 4(7) | 11(17) | 8(10) | 4(8) | 0(0) | 1(3) | 0(0) | **12** | **3** | **7** |
|  | **correct feed therapy*** | |  |  |  |  |  |  |  |  |  |  |
|  | follow-up | 10(13) | 4(7) | 11(17) | 8(10) | 3(6) | 0(0) | 1(3) | 0(0) | **12** | **2** | **7** |
| **Mortality (%)** | | |  |  |  |  |  |  |  |  |  |  |
|  | baseline | 7/15 (47) | 0/2 | 0/13 | 7/23 (30) | 1/3 (33) | 5/22 (23) | 1/18 (6) | 1/10 (10) | **19** | **18** | **19** |
|  | follow-up | 23/68 (34) | 5/54 (9) | 28/63 (44) | 20/80 (25) | 8/45 (18) | 9/31 (29) | 5/34 (15) | 4/16 (25) | **28** | **22** | **25** |

*Inadequate data at baseline

^†^weighted pooled group summaries
